# Administration of an antagonist of P2X7 receptor to EAE rats prevents a decrease of expression of claudin-5 in cerebral capillaries

**DOI:** 10.1007/s11302-018-9620-9

**Published:** 2018-08-08

**Authors:** Tomasz Grygorowicz, Beata Dąbrowska-Bouta, Lidia Strużyńska

**Affiliations:** Laboratory of Pathoneurochemistry Department of Neurochemistry, Mossakowski Medical Research Centre, Polish Academy of Sciences, 5 Pawińskiego str., 02-106 Warsaw, Poland

**Keywords:** Multiple sclerosis, Purinergic receptors, Pericytes, Blood-brain barrier, Tight junction proteins

## Abstract

Purinergic P2X receptors, when activated under pathological conditions, participate in induction of the inflammatory response and/or cell death. Both neuroinflammation and neurodegeneration represent hallmarks of multiple sclerosis (MS), an autoimmune disease of the central nervous system. In the current study, we examined whether P2X7R is expressed in brain microvasculature of rats subjected to experimental autoimmune encephalomyelitis (EAE) and explore possible relationships with blood-brain barrier (BBB) protein—claudin-5 after administration of P2X7R antagonist—Brilliant Blue G (BBG). Capillary fraction isolated from control and EAE rat brains was subjected to immunohistochemical and Western blot analyses. We document the presence of P2X7R in brain capillaries isolated from brain tissue of EAE rats. P2X7R is found to be localized on the abluminal surface of the microvessels and is co-expressed with PDGFβR, a marker of pericytes. We also show over-expression of this receptor in isolated capillaries during the course of EAE, which is temporally correlated with a lower protein level of PDGFβR, as well as claudin-5, a tight junction-building protein. Administration of a P2X7R antagonist to the immunized rats significantly reduced clinical signs of EAE and enhances protein expression of both claudin-5 and PDGFβR. These results indicate that P2X7 receptor located on pericytes may contribute to pathological mechanisms operated during EAE in cerebral microvessels influencing the BBB integrity.

## Introduction

During neuroinflammation, activated or injured cells release high levels of extracellular ATP which act via specific metabotropic (P2Y) or ionotropic (P2X) groups of receptors. One of the representatives of the latter group is P2X7 receptor (P2X7R), which is among the most abundant receptors in the central nervous system (CNS). The most significant P2X7R-mediated response is activation of the inflammasome and further maturation and release of interleukin-1β (IL-1β), a potent proinflammatory cytokine which triggers the inflammatory cascade [1–3]. Apart from a release of inflammatory mediators, over-activation of this receptor may also produce a release of excitatory neurotransmitters such as glutamate [4] or lead to the formation of plasma membrane pores and subsequent cell death [5].

Therefore, this type of purinergic receptor evokes significant interest in context of inflammatory/neurodegenerative changes observed in various CNS disorders [6], including multiple sclerosis (MS) [7–9].

MS is an autoimmune neuroinflammatory disease which leads to progressive physical and cognitive disability. It is characterized by inflamed plaques of demyelination in the white matter, oligodendroglial cell death, and damage of axons. Cells present in plaques (immune cells and glial cells) exacerbate inflammation by secreting proinflammatory cytokines. One of the characteristic pathological features of the disease is the presence of T cells in the perivascular area which infiltrate the CNS from the peripheral circulation [10]. Infiltration of the CNS by immune cells is associated with increased permeability of the blood-brain barrier (BBB) under inflammatory conditions which is observed at a very early stage of the disease. Studies performed on an animal model of MS suggest the existence of a strong correlation between BBB dysfunction and the severity of the disease [11, 12]. Additionally, increased permeability of BBB has been linked to loss or delocalization of endothelial tight junction proteins in brain specimens obtained from MS patients [13, 14].

Morphologically the BBB is constructed of endothelial cells connected by tight junctions. However, basement membrane, pericytes, and astrocytes also play important roles in maintaining integrity of the BBB and collectively form the neurovascular unit. It is important to note that interactions between endothelial cells and pericytes are crucial for proper function of blood microvessels [15, 16].

Extracellular nucleotides and different groups of purinergic receptors have been demonstrated to play significant roles in MS pathology [17]. P2X7R may be involved in MS pathology as it has been identified both in patients and animals subjected to experimental autoimmune encephalomyelitis (EAE). Over-expression of this receptor has been observed in oligodendrocytes [18], neurons, and astrocytes [8, 9] and is always accompanied by cell activation, dysfunction, or damage. However, the changes in P2X7R expression in rat brain microvasculature during the course of EAE as well as possible effects on tight junctional proteins have not been studied.

Thus, in the current work, we demonstrate the presence of P2X7R on pericytes of cerebral microvessels of rats subjected to EAE, the temporal profile of P2X7R, PDGFβR, and claudin-5 expression during the course of the disease, as well as the protective effect provided by administration of Brilliant Blue G (BBG; an antagonist of P2X7R) on the expression of these proteins.

We investigated capillaries isolated from brain, although MS/EAE is traditionally viewed as a CNS white matter disease with inflammatory lesions most often located in the spinal cord. However, a lot of research have contributed to refinement of our understanding of this pathology, indicating that it applies equally to the spinal cord and brain [9, 19–22].

## Materials and methods

### Animals and immunization procedure

Female Lewis rats weighing 180–210 g were obtained from the Animal House at the Mossakowski Medical Research Center, Polish Academy of Sciences (Warsaw, Poland). The rats were housed in a temperature- and humidity-controlled environment and had free access to water and standard laboratory fodder. All procedures were performed according to the EU Directive for Animal Use in Experiments and were approved by the Local Care and Use of Experimental Animal Committee (approval no. 48/2011).

Rats were randomly assigned to four groups (control, EAE, EAE + BBG, EAE + saline). The experimental autoimmune encephalomyelitis (EAE group) was induced as described previously [9, 23] by subcutaneous injections of 100 μL of an inoculum containing homogenate of guinea pig spinal cord in PBS, Freund’s complete adjuvant (CFA; Difco, Detroit, MI), and 2 mg/mL of *Mycobacterium tuberculosis* (Difco H37RA, Detroit, MI) into each hind foot. The rats were weighed daily and development of neurological symptoms was observed. Neurological deficits were scored according to the following scale: 0—no symptoms, 1—loss of tail tone, 2—tail and hind limb weakness, 3—hind limb paralysis, 4—ascending paralysis (paraplegia), and 5—moribund state or death [24]. The control group of rats (control) was not immunized. In the BBG-treated group (EAE + BBG), a Brilliant Blue G, a specific antagonist of P2X7R, was administered daily to EAE rats at a dose of 50 mg/kg b.w. starting from day 0 until day 6 postimmunization via a catheter implanted into the internal jugular vein. The vehicle-treated group (EAE + saline) received saline chloride instead of BBG. Animals from the control and immunized groups were sacrificed on different days postimmunization (2, 4, 6, 8, 12 d.p.i.), whereas the BBG/NaCl-treated rats were sacrificed in the asymptomatic (4 d.p.i.) and symptomatic phases (12 d.p.i.) of the disease. After decapitation, the brains were rapidly removed and capillary fractions were isolated.

### Preparation of the capillary fraction

**Fig. 1:**
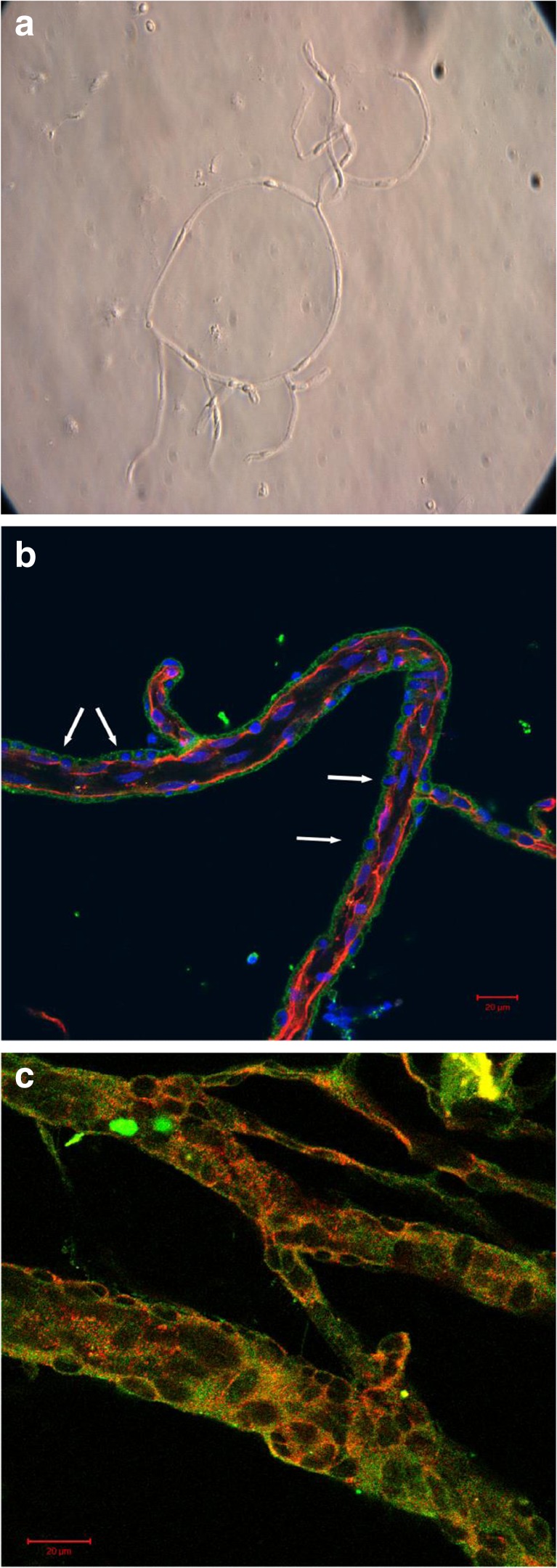
**a** Typical light microscopic image of cerebral capillaries isolated from control rat brain. Magnification ×20. **b** Localization of P2X7R in capillaries isolated from control rat brain. Triple immunofluorescent staining, P2X7R—green, claudin-5—red (BBB marker), Hoechst 33342 dye (DNA marker)—blue. P2X7R-specific green fluorescence is observed on the abluminal surface of microvessels in the cellular layer with round nuclei (arrows). Bar 20 μm. **c** Co-localization of P2X7R and PDGFβR in capillaries isolated from EAE rat brain. Double immunofluorescent staining: P2X7R—green, PDGFβR—red, merge—yellow. Bar 20 μm

The capillary fraction was isolated from gray matter of rat hemispheres according to the method described by Mrsulja et al. [25]. Briefly, gray matter prepared from freshly isolated brains (two brains per sample) was homogenized in Ringer’s solution and centrifuged at 1500×*g*, for 10 min at 4 °C. The pellet was re-suspended in the same buffer, and centrifugation was repeated two times under the same conditions. The final pellet was homogenized in 10 mL of 0.25 M sucrose and centrifuged in a discontinuous sucrose gradient (0.25:1:1.5 M sucrose) (30,000×*g*, 30 min, 4 °C). The fraction containing microvessels obtained at the bottom of the tube was further subjected to other procedures. The purity of the microvessel fraction and the efficiency of preparation was monitored with brief observations under a light microscope (Zeiss Axiovert 25) (Fig. 1a). Samples of the capillary fraction were frozen and stored for Western blot analysis or smeared on a slide for immunohistochemical analysis.

### Immunohistochemical procedures

Microvessels smeared on a slide were stained using primary antibody anti-claudin-5 (Invitrogen Corp., Carlsbad, CA, USA, 1:500), anti-P2X7R (Alomone Labs, Jerusalem, Israel, 1:200), or anti-platelet derived growth factor β receptor (PDGFβR) (Abcam, 1:1000). The samples were then stained with secondary antibody conjugated with AlexaFluor 546 or AlexaFluor 488 (Invitrogen Corp., Carlsbad, CA, USA, 1:100). Nuclei were stained with Hoechst 33342 dye (Sigma-Aldrich). Analyses of specimens was performed using a LSM 780 Zeiss confocal microscope with the Zen 2011 software system. Figures were created using Corel Draw X3.

### Western blot analysis

Protein concentrations in microvessel fractions were measured according to the method of Lowry et al. [26] using bovine albumin as a standard.

Microvessel fractions were subjected to SDS-polyacrylamide gel (10%) electrophoresis (SDS-PAGE). Samples containing 50 μg of protein were separated and transferred onto a nitrocellulose membrane. Membranes were blocked using 4% fat free milk for 30 min followed by overnight incubation at 4 °C with primary antibodies: anti-P2X7R (Alomone Labs, Jerusalem, Israel; 1:200), anti-PDGFβR (Abcam, Cambridge, UK; 1:500), or anti-claudin-5 (Invitrogen Corp., Carlsbad, CA, USA; 1:500). Then, the membrane was incubated with the secondary antibody conjugated with HRP (Sigma-Aldrich, 1: 10000). Bands were visualized using the ECL kit (Amersham) and Hyperfilm ECL. Blots were digitized using Image Scanner III (GE Healthcare) and densitometry was performed with the ImageJ program (Wayne Rasband, National Institutes of Health, USA).

### Statistical analysis

Results are presented as means ± SD from three to four experiments. Inter-group comparisons of densitometric measurements of immunoblots was performed using one-way analysis of variance (ANOVA) followed by the post hoc Dunnett’s test.

## Results

### P2X7R is located on pericytes of the capillary fraction isolated from control and EAE rat brain

Immunoreactivity of P2X7R was observed in isolated microvessels (Fig. 1a) after double immunostaining with antibody against P2X7R and claudin-5, a tight junction protein and accepted marker of endothelial cells. The pattern of distribution of this protein was found to be consistent with continuous junctional complexes between endothelial cells. Claudin-5-positive endothelial cells were found to form a deeper layer of the vessel, whereas P2X7R-positive cells were observed on the abluminal surface of microvessels. The superficial position of the P2X7R-immunostained cells suggests that the receptor is located on pericytes. The characteristic round morphology of nuclei of P2X7R-positive cells supports this observation (Fig. 1b). To confirm that P2X7R is localized on pericytes, these cells were contacted with an antibody against PDGFβR, which is a specific marker of pericytes. Double immunostaining shows co-localization of immunoreactivity against P2X7R and PDGFβR (Fig. 1c).

### The course of the disease in immunized rats: the effect of BBG, an antagonist of P2X7R

Immunized female Lewis rats were observed to gain weight and develop neurological deficits characteristic of EAE as described in detail previously [9, 23]. The first symptoms (paralysis of tail and hind limbs), which were assessed using the five-score scale, were observed to appear at days 9–10 postimmunization (p.i.). Subsequently, the symptoms peaked at days12–13 p.i., followed by recovery at day 18 p.i.

Administration of BBG was found to delay the onset of the disease and the peak of symptoms by about 2 days relative to both the EAE and vehicle control (EAE + NaCl) rats. A reduction in the severity of neurological deficits was also observed. The maximal disease score was reduced from 3.0 (EAE) and 2.5 (EAE + NaCl) to 1–1.5 (EAE + BBG) (Table 1).

**Table 1:** Clinical parameters of animals subjected to EAE and animals simultaneously treated with P2X7R antagonist (BBG) or vehicle (NaCl)

Parameter	EAE	EAE + BBG	EAE + NaCl
Body weight in symptomatic phase (g)	130 ± 0.8	165 ± 0.5*^, #^	135 ± 0.7
Maximal CI (score)	3 ± 0.5	1.5 ± 0.5*^, #^	2.5 ± 0.8
Inductive phase (days)	10 ± 1.3	12 ± 1.1	10 ± 1.4

The values represent the means ± SD from 12 animals in the EAE group and 8 animals in the EAE + BBG/NaCl group

CI cumulative index

*p < 0.05 values significantly different from EAE, ^#^*p* < 0.05 values significantly different from EAE + NaCl group

### Time course of changes in immunoreactivity of P2X7R, PDGFβR, and claudin-5 in capillaries isolated from brain of EAE rats and effect of administration of the P2X7R antagonist

**Fig. 2:**
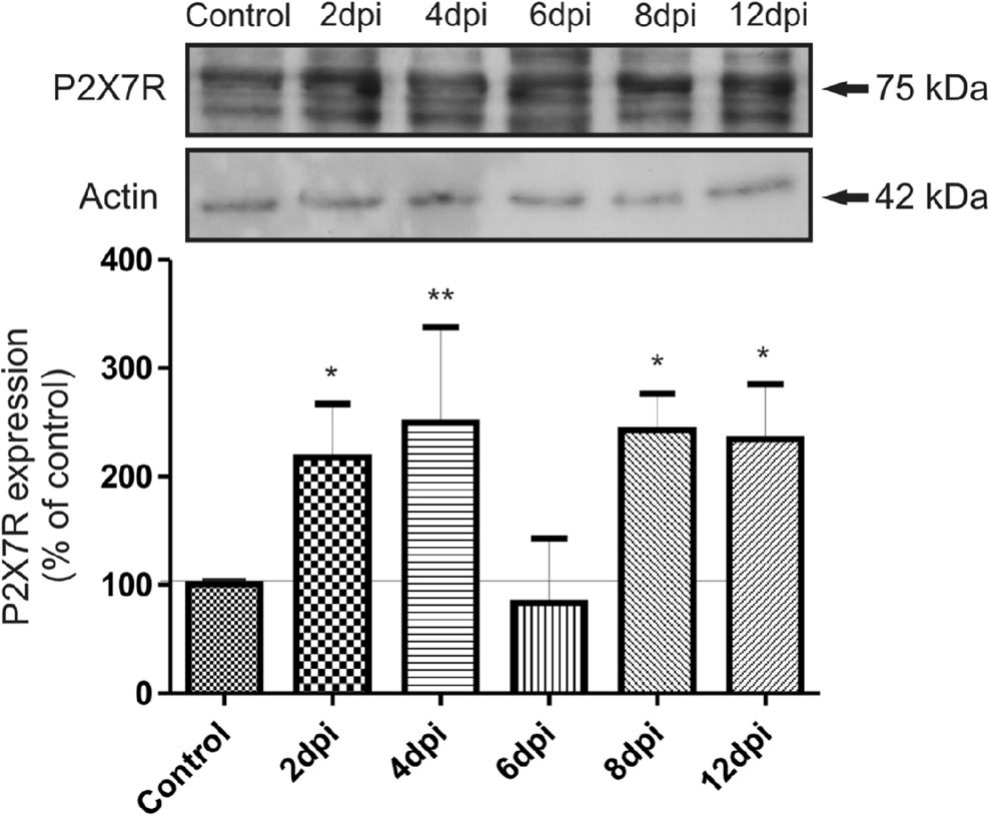
Relative protein concentration of P2X7R in cerebral capillaries isolated from control and EAE rats at different time points postimmunization (d.p.i.), representative immunoblots and graph illustrating the mean results of densitometric measurements of four independent immunoblots performed using four distinct animals per group. The relative density was measured against β-actin as an internal standard and expressed as a percentage of non-immunized control. *p < 0.05, **p < 0.01 vs. control (one-way ANOVA with post hoc Dunnett’s test)

**Fig. 3:**
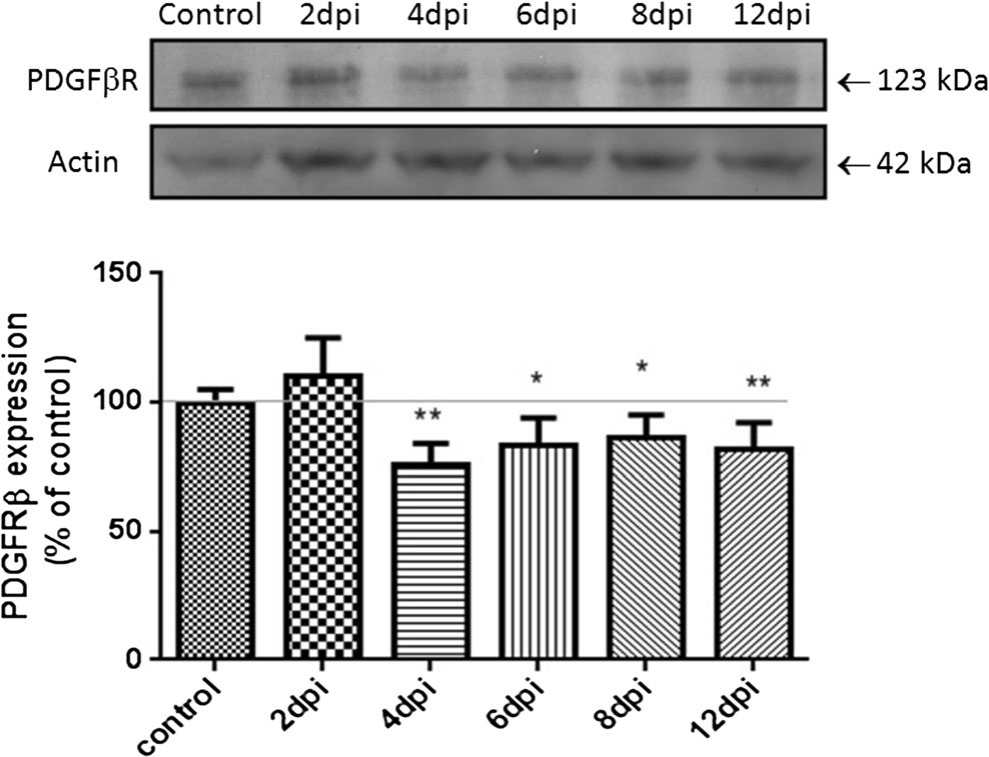
Relative protein concentration of PDGFβR in cerebral capillaries isolated from control and EAE rats at different time points postimmunization (d.p.i.), representative immunoblots and graph illustrating the mean results of densitometric measurements of four independent immunoblots performed using four distinct animals per group. The relative density was measured against β-actin as an internal standard and expressed as a percentage of non-immunized control. *p < 0.05, **p < 0.01 vs. control (one-way ANOVA with post hoc Dunnett’s test)

**Fig. 4:**
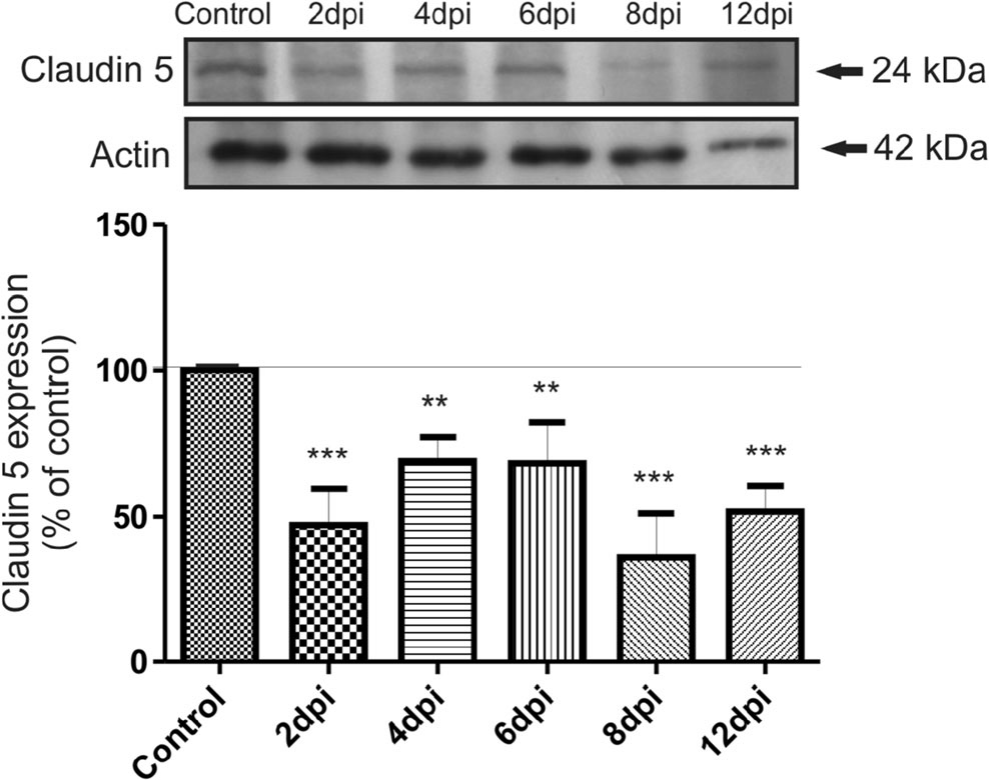
Relative protein concentration of claudin-5 in cerebral capillaries isolated from control and EAE rats at different time points postimmunization (d.p.i.), representative immunoblots and graph illustrating the mean results of densitometric measurements of four independent immunoblots performed using four distinct animals per group. The relative density was measured against β-actin as an internal standard and expressed as a percentage of non-immunized control. **p < 0.01, ***p < 0.001 vs. control (one-way ANOVA with post hoc Dunnett’s test)

**Fig. 5:**
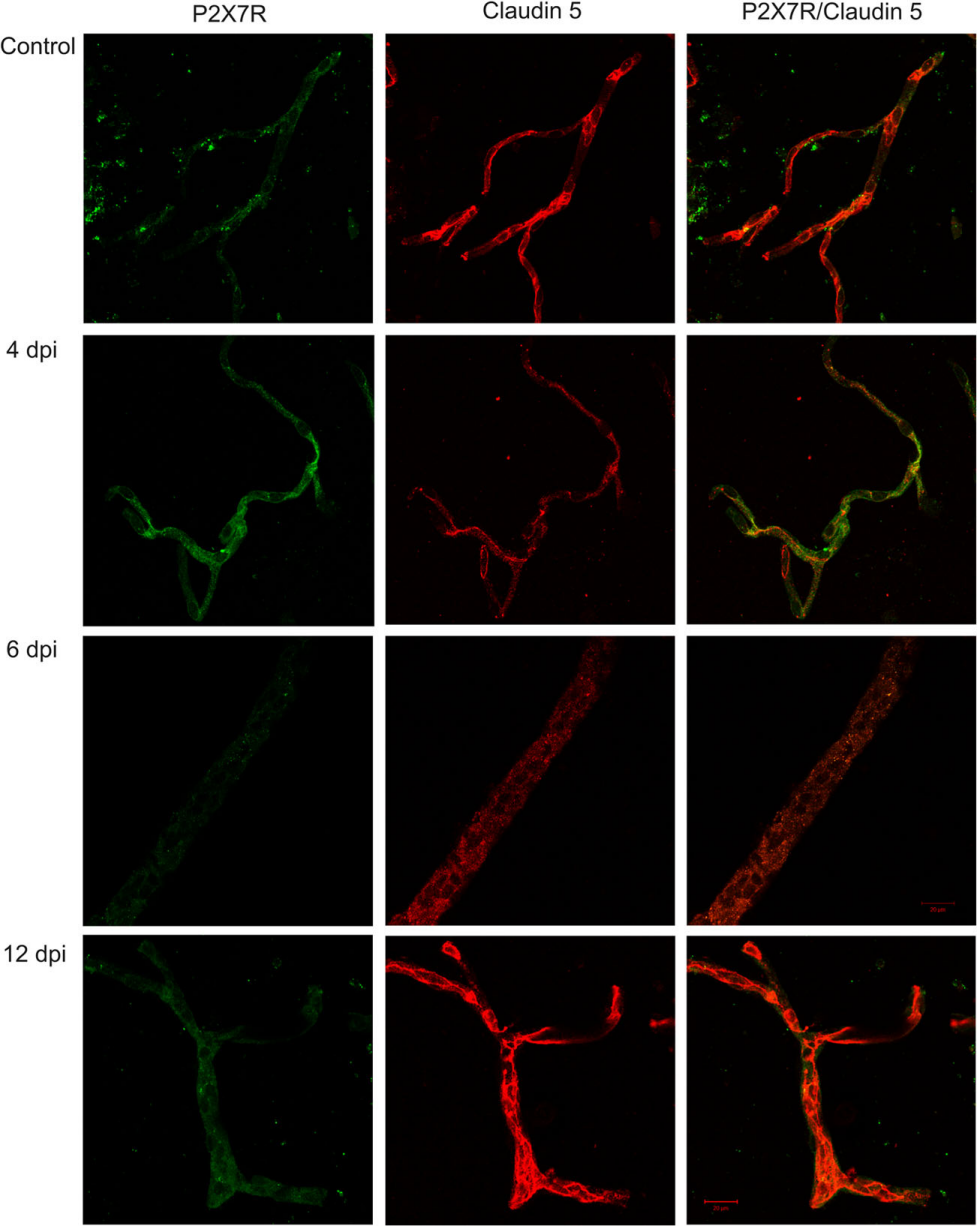
P2X7R- and claudin-5-specific immunofluorescence in the capillary fraction obtained from control and EAE rat brains in different phases of the disease (4, 6, and 12 d.p.i.). Double immunofluorescent staining: green—P2X7R, red—claudin-5. Bar 20 μm

We observed changes in P2X7R, PDGFβR, and claudin-5 expression in microvessels during the course of EAE. Western blots revealed a significant increase in P2X7R protein concentration along with development of the disease (Fig. 2) exceeding the control value by an average of 123% at 2, 4, 8, and 12 d.p.i. (*p* < 0.05 − *p* < 0.01 vs. control). Exclusively at day 6 p.i., we did not observe elevation of P2X7R protein. Instead, P2X7R protein levels were found to decrease below those of the control group. Concomitantly, relative protein level of PDGFβR tend to decrease by about 20–25% relative to control rats (*p* < 0.05 − *p* < 0.01), starting from the fourth day p.i. (Fig. 3). We further investigated whether expression of claudin-5 protein is altered in parallel with changes in protein levels of P2X7R and PDGFβR during development of EAE. We observed decreased levels of claudin-5 protein ranging from 36 to 69% of control values (*p* < 0.01 − *p* < 0.001) at all of the time points (Fig. 4). Analysis of immunohistochemical staining of capillary fractions for P2X7R/claudin-5 (Fig. 5) indicates a similar pattern of changes, i.e., decreased immunoreactivity of tight junction proteins and increased immunoreactivity of P2X7R protein during EAE relative to non-immunized animals.

**Fig. 6:**
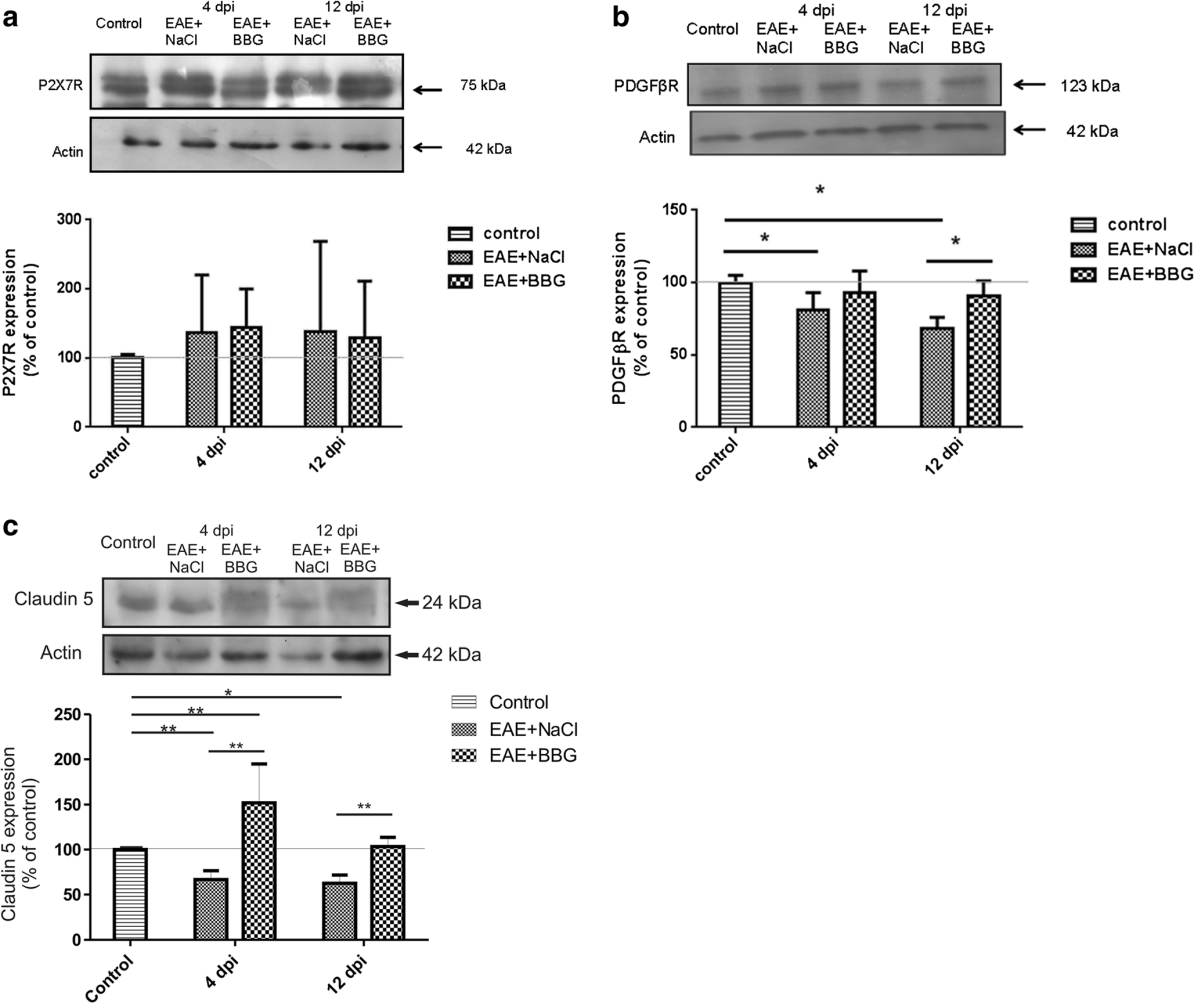
The effect of BBG administration on the expression of P2X7R, PDGFβR, and claudin-5. Relative protein concentration of P2X7R (**a**), PDGFβR (**b**), and claudin-5 (**c**) in cerebral capillaries isolated from control, EAE rats, and BBG-treated EAE rats in asymptomatic (4 d.p.i.) and symptomatic (12 d.p.i.) phases of the disease. Representative immunoblots and graphs illustrating the mean results of densitometric measurements of four independent immunoblots performed using four distinct animals per group and expressed as a percentage of non-immunized control. The relative density was measured against β-actin as an internal standard. *p < 0.05, **p < 0.01 vs. control or vehicle-treated EAE rats (one-way ANOVA with post hoc Dunnett’s test)

Administration of BBG did not influence significantly protein expression of P2X7R (Fig. 6a) but recovered the level of PDGFβR in the symptomatic phase (12 d.p.i.) (Fig. 6b). Western blot analysis of PDGFβR in microvessels of rats subjected to EAE and treated simultaneously with BBG revealed prevention of the expected decrease of PDGFβR expression by about 20% (*p* < 0.05) relative to vehicle-treated animals. Furthermore, selective inhibition of P2X7R was found to influence the observed profile of changes in endothelial junction protein. In the asymptomatic phase (4 d.p.i.), the protein concentration of claudin-5 was found to be significantly (*p* < 0.01) higher in BBG-treated rats than in vehicle-treated EAE rats by about 70%. In the symptomatic phase (12 d.p.i.), protein expression was observed to recover to the control value but was still higher than its value in the vehicle-treated EAE animals (*p* < 0.01) (Fig. 6c).

## Discussion

### Microvascular expression of P2X7R during the course of EAE

P2X7R is widely distributed in brain in neurons and glial cells (astrocytes, oligodendrocytes, and microglia) [27, 28]. Its presence has also been confirmed in retinal microvasculature, wherein ATP has been shown to regulate the contraction of isolated rat retinal microvessels through the activation of P2X7R [29]. In cerebral microvessels, P2X7R has been reported to be over-expressed in endothelial cells after experimental intracerebral hemorrhage [30]. Results of the current study using a capillary fraction isolated from both control and EAE rat brains clearly showed that this receptor is expressed on pericytes rather than on endothelial cells. The P2X7R-specific immunofluorescence was found to be present in the cellular layer with round-shaped nuclei on the abluminal surface of endothelial cells indicating localization on pericytes. Pericyte-related expression of this receptor was confirmed by using an antibody against PDGFβR, which is known to be a useful marker of these cells [31]. To our knowledge, P2X7R has not yet been reported to be present in pericytes of cerebral microvessels, although its presence was confirmed in pericyte-containing microvessels isolated from rat retina [29].

Evidence indicates that P2X7R is involved in a vast majority of central nervous system (CNS) pathologies, including MS (for a review, see [32]). Development of clinical signs of EAE as well as axonal damage and activation of astroglia were found to be significantly reduced in P2X7R-deficient mice [33] and BBG-administered rats [9]. However, it has not been determined whether P2X7R is present on cerebral microvessels during MS/EAE. The time course of P2X7R expression in capillaries isolated from the rat brains along with EAE development indicates that its expression increases early after immunization (2 d.p.i.) and persists at elevated levels in the symptomatic phase. The decrease of P2X7R levels at 6 d.p.i. is an interesting observation. One possible explanation is activation of cellular mechanisms negatively regulating the expression of the protein in this phase of the disease. However, pericytes are the cells that express P2X7R, and thus, any reduction in the number of pericytes could be the cause of lower protein levels. The decreased number of pericytes, preceded by high over-expression of P2X7R at 2–4 d.p.i., may be the result of cytotoxic activity of P2X7R. Such explanation might be supported by visible decrease of relative expression of PDGFβR which is regarded as a marker of these cells.

Alternatively, it may be the effect of migration of pericytes from the microvessel walls to the surrounding tissue [34]. It has been reported that the loss of pericytes is correlated with pathology of diseases such as stroke, brain tumors, and diabetic retinopathy [35] leading to local disintegration of the BBB [36]. This topic warrants further studies, because pericytes play a crucial role in communication with other cells of the neurovascular unit via physical contact as well as autocrine and paracrine regulation [31]. In endothelial cells (EC), pericytes have been reported to induce synthesis of occludins and claudins through the release of angiopoetin-1 [37]. Moreover, PDGFβR-deficient mice exhibit the reduction of tight junction proteins such as ZO-1, occluding and claudin-5 [38], as well as increased vascular permeability due to enhanced transcytosis and tight junction abnormalities [16]. These data indicate that undisturbed communication between pericytes and ECs allows proper function of the BBB.

### Possible involvement of pericytic P2X7R in disintegration of tight junction complexes during EAE

As mentioned above, the main role of pericytes is to regulate proper functioning of the BBB by integrating endothelial and astrocytic functions at the level of the neurovascular unit [16]. The integrity of cerebral microvessels depends mainly on the tightness of junction complexes between endothelial cells. Thus, disturbances in protein complexes and decreased expression may lead to disintegration of the BBB and increased permeability of vessels [39].

In the context of our findings that P2X7R protein expression increases in EAE microvessels, in parallel with decreased PDGFβR, starting from the early asymptomatic phase of the disease, we sought to determine if it would be connected with changes in protein markers of EC. The level of claudin-5 was analyzed, since it has been shown to be connected with the integrity of the BBB (for a review, see [40]) as one of the most important tight junctional proteins. Loss of claudin-5 actively contributes to severe BBB dysfunction and increased permeability of vessels [41]. A significant decrease in protein concentration of claudin-5 was found to coincide temporarily with both increased expression of P2X7R and diminished expression of pericytic PDGFβR. This temporal correlation between three proteins: pericytic P2X7R, pericytic PDGFβR, and endothelial claudin-5, appears to be significant because it suggests that pericyte-EC interactions may be partially mediated by P2X7R. Indeed, it appears that administration of a selective P2X7R antagonist to EAE rats partially recovered the level of PDGFβR and has a protective effect, considerably increasing expression of claudin-5 protein in an early asymptomatic phase. The results are in agreement with those of Zhao and coworkers [30], who have shown that administration of the selective P2X7R inhibitor A438079 upregulates the expression of occludin in rats subjected to experimental intracerebral hemorrhage. Moreover, inhibition of P2X7R by BBG has been found to partially alleviate neurological deficits in EAE rats. This observation is consistent with our previous results [9]. It has been reported that the severity of the neurological symptoms has a strong correlation with dysfunctional BBB [11, 12].

This indicates that there is direct or indirect regulation of this junctional protein through the P2X7R-dependent pathways. As evidenced recently, RhoA kinase may directly contribute to the P2X7R-induced disruption of the BBB during experimental hemorrhage [30]. Indirect regulation under inflammatory conditions in MS/EAE may occur through IL-1β and other proinflammatory cytokines [3] which are released by immune cells, astroglia, and microglia in a P2X7R-dependent manner (for a review, see [27]) affecting the BBB [10]. Interleukin IL-1β may indirectly destabilize the BBB by enhancing expression of metalloproteinase-9 (MMP-9) [42], which is involved in proteolysis of tight junction proteins such as occludins and claudin-5 [10, 43].

In conclusion, we demonstrate that P2X7R is present on cerebral microvessels of control and EAE rats and its expression is connected with pericytes. Starting from the very early phase of EAE, this receptor is over-expressed and this is accompanied by downregulation of expression of PDGFβR and claudin-5. An important finding from our study is the demonstration that blockage of P2X7R preserves this effect, thereby suggesting that it may be considerable involvement of this receptor located on pericytes in disintegration of the BBB during EAE.

## References

[1] Pelegrin P, Surprenant A (2006). Pannexin-1 mediates large pore formation and interleukin-1beta release by the ATP-gated P2X7 receptor. EMBO J.

[2] Lister MF, Sharkey J, Sawatzky DA, Hodgkiss JP, Davidson DJ, Rossi AG, Finlayson K (2007). The role of the purinergic P2X7 receptor in inflammation. J Inflamm (Lond).

[3] Mingam R, De Smedt V, Amedee T, Bluthe R-M, Kelley KW, Dantzer R, Laye S (2008). In vitro and in vivo evidence for a role of the P2X7 receptor in the release of Il-1 beta in the murine brain. Brain Behav Immun.

[4] Papp L, Vizi ES, Sperlágh B (2007). P2X7 receptor mediated phosphorylation of p38MAP kinase in the hippocampus. Biochem Biophys Res Commun.

[5] Volonté C, Apolloni S, Skaper SD, Burnstock G (2012). P2X7 receptors: channels, pores and more. CNS Neurol Disord Drug Targets.

[6] Takenouchi T, Sekiyama K, Sekigawa A, Fujita M, Waragai M, Sugama S, Iwamaru H, Hashimoto M (2010). P2X7 receptor signaling pathway as a therapeutic target for neurodegenerative diseases. Arch Immunol Ther Exp.

[7] Matute C (2008). P2X7 receptors in oligodendrocytes: a novel target for neuroprotection. Mol Neurobiol.

[8] Grygorowicz T, Strużyńska L, Sulkowski G, Chilimoniuk M, Sulejczak D (2010). Temporal expression of P2X7 purinergic receptor during the course of experimental autoimmune encephalomyelitis. Neurochem Int.

[9] Grygorowicz T, Wełniak-Kamińska M, Strużyńska L (2016). Early P2X7R-related astrogliosis in autoimmune encephalomyelitis. Mol Cell Neurosci.

[10] Alvarez JI, Cayrol R, Prat A (2011). Disruption of central nervous system barriers in multiple sclerosis. Biochim Biophys Acta.

[11] Fabis MJ, Scott GS, Kean RB, Koprowski H, Hooper DC (2007). Loss of blood-brain barrier integrity in the spinal cord is common to experimental allergic encephalomyelitis in knockout mouse models. Proc Natl Acad Sci U S A.

[12] Morgan L, Shah B, Rivers LE, Barden L, Groom AJ, Chung R, Higazi D, Desmond H, Smith T, Staddon JM (2007). Inflammation and dephosphorylation of the tight junction protein occludin in an experimental model of multiple sclerosis. Neurosci.

[13] Kirk J, Plumb J, Mirakhur M, McQuaid S (2003). Tight junctional abnormality in multiple sclerosis white matter affects all calibres of vessel and is associated with blood-brain barrier leakage and active demyelination. J Pathol.

[14] Leech S, Kirk J, Plumb J, McQuaid S (2007). Persistent endothelial abnormalities and blood-brain barrier leak in primary and secondary progressive multiple sclerosis. Neuropathol Appl Neurobiol.

[15] Armulik A, Abramsson A, Betsholtz C (2005). Endothelial/pericyte interactions. Circ Res.

[16] Armulik A, Genové G, Mäe M, Nisancioglu MH, Wallgard E, Niaudet C, He L, Norlin J, Lindblom P, Strittmatter K, Johansson BR, Betsholtz C (2010). Pericytes regulate the blood-brain barrier. Nature.

[17] Cieślak M, Kukulski F, Komoszyński M (2011). Emerging role of extracellular nucleotides and adenosine in multiple sclerosis. Purinergic Signal.

[18] Matute C, Torre I, Perez-Cerda F, Perez-Samartin A, Alberdi E, Etxebarria E, Arranz AM, Ravid R, Rodrigez-Antigüedad A, Sanchez-Gomez MV, Domercq M (2007). P2X7 receptor blockade prevents ATP excitotoxicity in oligodendrocytes and ameliorates experimental autoimmune encephalomyelitis. J Neurosci.

[19] Ayers MM, Hazelwood LJ, Catmull DV, Wang D, McKormack Q, Bernard CC, Orian JM (2004). Early glial responses in murine models of multiple sclerosis. Neurochem Int.

[20] Wang D, Ayers MM, Catmull DV, Hazelwood LJ, Bernard CC, Orian JM (2005). Astrocyte-associated axonal damage in pre-onset stages of experimental autoimmune encephalomyelitis. Glia.

[21] Brown DA, Sawchenko PE (2007). Time course and distribution of inflammatory and neurodegenerative events suggest structural bases for the pathogenesis of experimental autoimmune encephalomyelitis. J Comp Neurol.

[22] Sulkowski G, Dąbrowska-Bouta B, Salińska E, Strużyńska L (2014). Modulation of glutamate transport and receptor binding by glutamate receptor antagonists in EAE rat brain. PLoS One.

[23] Sulkowski G, Dąbrowska-Bouta B, Chalimoniuk M, Strużyńska L (2013). Effects of antagonists of glutamate receptors on pro-inflammatory cytokines in the brain cortex of rats subjected to experimental autoimmune encephalomyelitis. J Neuroimmunol.

[24] Kerschensteiner M, Stadelmann C, Buddeberg BS, Merkler D, Bareyre FM, Anthony DC, Linington C, Bruck W, Schwab ME (2004). Targeting experimental autoimmune encephalomyelitis lesions to a predetermined axonal tract system allows for refined behavioral testing in an animal model of multiple sclerosis. Am J Pathol.

[25] Mrsulja BB, Mrsulja BJ, Fujimoto T, Klatzo I, Spatz M (1976). Isolation of brain capillaries: a simplified technique. Brain Res.

[26] Lowry OH, Rosebrough NJ, Farr AL, Randall RJ (1951). Protein measurement with the Folin phenol reagent. J Biol Chem.

[27] Sperlagh B, Vizi ES, Wirkner K, Illes P (2006). P2X7 receptors in the nervous system. Prog Neurobiol.

[28] Yu Y, Ugawa S, Ueda T, Ishida Y, Inoue K, Kyaw Nyunt A, Umemura A, Mase M, Yamada K, Shimada S (2008). Cellular localization of P2X7 receptor mRNA in the rat brain. Brain Res.

[29] Kawamura H, Sugiyama T, Wu DM, Kobayashi M, Yamanishi S, Katsumura K, Puro DG (2003). ATP: a vasoactive signal in the pericyte-containing microvasculature of the rat retina. J Physiol.

[30] Zhao H, Zhang X, Dai Z, Feng Y, Li Q, Zhang JH, Liu X, Chen Y, Feng H (2016). P2X7 receptor suppression preserves blood-brain barrier through inhibiting RhoA activation after experimental intracerebral hemorrhage in rats. Sci Rep.

[31] Armulik A (2011). Pericytes: developmental, physiological and pathological perspectives, problems and promises. Dev Cell Rev.

[32] Sperlagh B i PI (2014). P2X7 receptor: an emerging target in central nervous system diseases. Trends Pharmacol Sci.

[33] Sharp AJ, Polak PE, Simonini V, Shao XL, Richardson JC, Bongarzone ER, Feinstein DL (2008). P2X7 deficiency suppresses development of experimental autoimmune encephalomyelitis. J Neuroinflamm.

[34] Dore-Duffy P, Owen C, Balabanov R, Murphy S, Beaumont T, Rafols JA (2000). Pericyte migration from the vascular wall in response to traumatic brain injury. Microvasc Res.

[35] Bonkowski D, Katyshev V, Balabanov RD, Borisov A, Dore-Duffy P (2011). The CNS microvascular pericyte: pericyte-astrocyte crosstalk in the regulation of tissue survival. Fluids Barriers CNS.

[36] Dore-Duffy P, LaManna JC (2007). Physiologic angiodynamics in the brain. Antioxid Redox Signal.

[37] Hori S, Ohtsuki S, Hosoya K, Nakashima E, Terasaki TA (2007). Pericyte-derived angiopoietin-1 multimeric complex induces occludin gene expression in brain capillary endothelial cells through Tie-2 activation in vitro. J Neurochem.

[38] Bell RD, Winkler EA, Sagare AP, Singh I, LaRue B, Deane R, Zlokovic BV (2010). Pericytes control key neurovascular functions and neuronal phenotype in the adult brain and during brain aging. Neuron.

[39] Obermeier B, Daneman R, Ransohoff RM (2013). Development, maintenance and disruption of the blood-brain barrier. Nat Med.

[40] Luissint A-C, Artus C, Glacial F, Ganeshamoorthy K, Couraud P-O (2012). Tight junctions at the blood brain barrier: physiological architecture and disease-associated dysregulation. Fluids Barriers CNS.

[41] Nitta T, Hata M, Gotoh S, Seo Y, Sasaki H, Hashimoto N, Furuse M, Tsukita S (2003). Size-selective loosening of the blood-brain barrier in claudin-5–deficient mice. J Cell Biol.

[42] Sozen T, Tsuchiyama R, Hasegawa Y, Suzuki H, Jadhav V, Nishizawa S, Zhang JH (2009). Role of interleukin-1beta in early brain injury after subarachnoid hemorrhage in mice. Stroke.

[43] Bauer AT, Burgers HF, Rabie T, Marti HH (2010). Matrix metalloproteinase-9 mediates hypoxia-induced vascular leakage in the brain via tight junction rearrangement. J Cereb Blood Flow Metab.

